# An Evaluation of Admissions to an Old Age Psychiatry Ward: A Quality Improvement Project

**DOI:** 10.1192/bjo.2022.315

**Published:** 2022-06-20

**Authors:** Michael Cooper, Clare Langan, Christopher Haxton, Philip Andrew, Mohd Yazid

**Affiliations:** NHS Greater Glasgow and Clyde, Glasgow, United Kingdom

## Abstract

**Aims:**

It has been well publicised the pressures on inpatient bed capacity within mental health services in recent years. The RCPsych have stated in their publication ‘Exploring mental health inpatient capacity’ that bed occupancy has risen above their recommended 85% occupancy target in most areas. Waiting lists for beds have also grown. The aim of this project was to identify whether there could have been any extra community resources in place that could have prevented admissions to hospital within an older adult CMHT. This in turn reducing the demand on inpatient beds.

**Methods:**

Inclusion criteria: All patients from the older adult CMHT admitted to either an organic or functional mental health inpatient bed between the 1st September and 31st December 2021.

All patients who met the inclusion criteria were discussed as part of a panel consisting of members of the MDT who were involved in the patients' ongoing care. The panel discussed each patient and individually scored each admission on a scale of 1–5 (where 1 was deemed to be very avoidable and 5 completely unavoidable). Where an admission did not score a 5 we considered whether anything could have been in place to have prevented the admission.

**Results:**

Our search identified 21 patients who had been admitted to the respective old age psychiatry ward during our period of interest. The predominant diagnosis of these patients was vascular dementia (n = 5), followed by Alzheimer's disease (n = 3). Following our consensus panel discussion, we identified that the most common reason for admission to hospital was for management of behavioural and psychological symptoms of dementia (n = 10), followed by increasing patient vulnerability (n = 4) in the community. Carer stress was a theme in 2 admissions. Following panel discussion regarding potential avoidability of admission, we identified that 14 out of the total of 21 admissions scored a 5, 1 scored 4, 1 scored 3, and 5 scored 2.

**Conclusion:**

Behavioural and psychological symptoms of dementia continues to remain a significant clinical challenge and was the most common reason for admission in our patient cohort. The majority of admissions to hospital in our cohort were deemed unavoidable as a result. However, we identified that carer stress was a significant theme in 2 out of our 21 admissions, suggesting potential scope to implement services which may reduce carer stress and ultimately, prevent hospital admission.

